# Buckling and Post-Buckling Behaviour of a Carbon Fibre-Reinforced Polymer Stiffened Panel: A Numerical and Experimental Study

**DOI:** 10.3390/polym18091068

**Published:** 2026-04-28

**Authors:** Andrea Sellitto, Angela Russo, Mauro Zarrelli, Valeria Vinti, Luigi Trinchillo, Pierluigi Perugini, Aniello Riccio

**Affiliations:** 1 Department of Engineering, University of Campania “Luigi Vanvitelli”, Via Roma 29, 81031 Aversa, CE, Italy; angela.russo@unicampania.it (A.R.); aniello.riccio@unicampania.it (A.R.); 2 Institute for Polymers Composites and Biomaterials, Italian National Research Council, Piazzale Enrico Fermi, 80055 Portici, NA, Italy; mauro.zarrelli@cnr.it; 3 Avio S.P.A., Via Leonida Bissolati, 76, 00187 Rome, RM, Italy; valeria.vinti@avio.com (V.V.); luigi.trinchillo@avio.com (L.T.); pierluigi.perugini@avio.com (P.P.)

**Keywords:** carbon fibre-reinforced polymers (CFRPs), buckling and post-buckling, digital image correlation (DIC), finite element analysis (FEA), composite structures, geometric imperfections, Hashin’s failure criteria, structural instability

## Abstract

The buckling and post-buckling responses of carbon fibre-reinforced polymer (CFRP) structures are strongly affected by geometric imperfections, boundary conditions, and material nonlinearities, making their reliable numerical prediction challenging. This work presents an integrated experimental–numerical investigation of a stiffened CFRP panel subjected to compressive loading, with the aim of improving model validation in instability regimes. The experimental campaign combines full-field measurements obtained through digital image correlation with local strain data from strain gauges, adopting a back-to-back configuration to capture the strain reversal associated with global buckling. The experimental results are compared with nonlinear finite element simulations incorporating intralaminar damage based on Hashin’s failure criteria. A good agreement between the numerical and experimental results is observed in the pre-buckling and early post-buckling regimes. However, increasing discrepancies arise at higher load levels, mainly due to manufacturing imperfections and uncertainties in boundary conditions, which influence the onset and evolution of localized deformation. Statistical indicators are employed to quantitatively assess the correlation between the experimental and numerical responses. The analysis focuses on the key response parameters, including the load–displacement behaviour, out-of-plane displacements, strain evolution, and damage initiation, enabling a comprehensive comparison of experimental and numerical results. The results demonstrate the effectiveness of combining full-field and point-wise measurements for validating numerical models of composite structures. Furthermore, the study highlights the limitations of idealized modelling assumptions and provides insights into the sensitivity of CFRP structures to imperfections in post-buckling and failure regimes.

## 1. Introduction

Instability and post-instability phenomena play a fundamental role in the structural response of thin-walled, reinforced carbon fibre-reinforced polymer (CFRP) components subjected to compressive loading, particularly in aerospace and lightweight structural applications. Although composite materials offer high stiffness-to-weight ratios and excellent tailoring capabilities [1,2,3], their instability behaviour is intrinsically complex and strongly dependent on geometric imperfections, boundary conditions, and material nonlinearities, as typically observed in thin-walled structures. As a result, the reliable prediction of instability loads, deformation patterns, and damage onset remains a challenging task for numerical models [4,5,6].

Nonlinear finite element methods are widely used to analyse the instability response of composite structures, often incorporating progressive damage models to account for material degradation. However, the predictive accuracy of such models is highly sensitive to assumptions regarding the initial imperfections and loading conditions, which are difficult to define in advance. Experimental validation is therefore crucial, especially in the post-buckling regime, where small deviations from idealized conditions can lead to significant changes in the structural response [7,8]. Indeed, several experimental and numerical studies have investigated the compressive response of CFRP structures, focusing on the complex interactions between instability and failure modes [9,10]. In particular, stiffened composite panels can be subjected to global and local buckling phenomena, while the typical failure modes include both intralaminar (fibre and matrix failure) and interlaminar (delamination) damage [11,12]. These mechanisms are strongly influenced by geometric imperfections, boundary conditions, and the stacking sequence, making the accurate prediction of structural behaviour particularly complex.

Traditional experimental validation strategies typically rely on point-wise measurements, such as strain gauges or displacement transducers. While effective at capturing local responses, these techniques provide limited information about the global deformation mechanisms associated with instability, and may fail to fully describe complex deformation modes. Full-field measurement techniques offer a complementary approach, allowing for the visualization and quantification of global displacement and deformation distributions over large areas of a structure. When combined with local sensors, they provide a more comprehensive experimental basis for the evaluation of numerical models.

In the literature, experimental investigations are often carried out using simplified configurations, such as coupon [13,14,15] or bending-dominated setups (three- and four- point bending tests) [16,17,18]. Although these configurations are able to capture specific failure modes, they may fail to fully reproduce the complexity of the compressive instability phenomena in stiffened panels. Hence, the present work focuses on a full-scale stiffened panel subjected to compressive loading, combining full-field measurements with local strain gauge data.

Among full-field measurement techniques, digital image correlation (DIC) has been widely adopted in experimental mechanics due to its non-invasive nature and its ability to provide spatially resolved displacement and deformation fields over extended areas [19,20,21,22]. DIC allows for the measurement of complex deformation modes without physically interacting with the specimen by tracking the shift of a random surface pattern across subsequent image sequences, making it particularly suitable for buckling and post-buckling analysis. Unlike point sensors, DIC allows for the identification of global deformation patterns, localized deformation concentrations, and mode transitions associated with instability phenomena. When combined with conventional strain gauges, it provides a solid experimental basis for the validation of nonlinear numerical models [23,24,25,26]. Moreover, several applications can be found in the literature [27,28,29,30] focusing on the use of DIC in compressive and other mechanical tests, including CFRP specimens and other classes of materials, where it has been used to observe deformation patterns under different loading conditions. For these reasons, in the present study DIC is employed as a full-field measurement technique.

In this context, the present work investigates the instability and post-instability behaviour of a composite panel reinforced through an integrated experimental–numerical approach. An experimental campaign is conducted using full-field displacement and strain measurements in combination with strain gauges placed on opposite faces of the panel, allowing for the detection of strain reversal phenomena induced by global instability. The experimental results are used in combination with a nonlinear finite element model that incorporates intralaminar damage to obtain a more comprehensive representation of the investigated phenomena, with particular attention to the influence of imperfections and boundary effects on the structural response. Indeed, even though the investigation is carried out on a specific stiffened panel configuration, the findings, in terms of experimental–numerical framework and observed instability mechanisms, can be considered representative of a broader class of thin-walled composite structures.

The paper is structured as follows: in Section 2 and Section 3 the experimental and numerical setup are respectively introduced, while in Section 4 the results are presented and discussed.

## 2. Experimental Setup and Measurement Strategy

**Figure 1:**
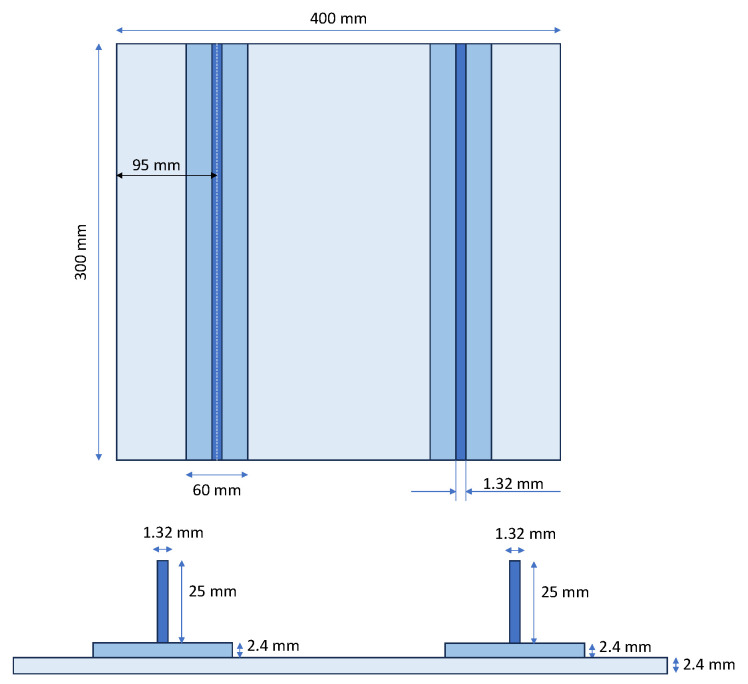
Geometrical description of the investigated panel.

The investigated test case consists of a double-stiffened panel made of carbon fibre-reinforced epoxy composite subjected to a compressive load. The stringers act as geometric reinforcement, significantly influencing the buckling and post-buckling responses of the structure. Figure 1 shows the panel’s geometry and dimensions, while Table 1 summarizes the material properties, which were experimentally derived by the authors in accordance with ASTM standards [31]. The stacking sequence of the panel is [−45,90,0,45]_2s_, while the stringer web and foot have stacking sequences of [0,90,90,0]_s_ and [(45,90,0,−45)_2s_, (0,90,90,0)_s_], respectively. The stacking sequences adopted for the stringer web and foot are representative of typical aeronautical stiffened composite subcomponents [32,33]; however, their selection is also supported by a preliminary numerical study (not reported here, as it is beyond the scope of this work) aimed at identifying configurations that enable the onset of instability phenomena within a target, non-excessive load range through an appropriate stiffness distribution between the skin and stringers. The stringers are bonded to the skin using an aeronautical structural adhesive (Araldite 2015-1 by Huntsman Corporation, Los Angeles, CA, USA). All plies are unidirectional, with a nominal thickness of 0.1 mm, and are made of a carbon fibre-reinforced epoxy system, specifically based on T800 carbon fibres and HXE23 epoxy resin.

The compressive test was carried out using an MTS Landmark hydraulic testing machine (Landmark MTS Model 370.50, MTS Systems Corporation, Eden Prairie, MN, USA) equipped with a 500 kN load cell. The applied load was continuously monitored by the machine loading cell throughout the test. In particular, the compressive test was carried out under load-controlled quasi-static conditions, with a loading rate of 0.5 kN/s. No specific ASTM standard was followed, as the test was performed on a structural component; the loading rate was selected to ensure stable acquisition of both DIC and strain gauge measurements while avoiding dynamic effects. Moreover, to characterize both the global deformation modes and local strain evolution, the experimental setup combined full-field measurements obtained via digital image correlation with local strain gauge measurements. The DIC measurements were performed on the back side of the panel to capture the out-of-plane displacements and in-plane strain fields, while strain gauges were installed on the front, where the stringers are located. The strain gauges were oriented both parallel (y-) and perpendicular (x-) to the applied load direction. This back-to-back configuration was intentionally adopted to capture the typical strain inversion induced by global buckling. The opposite strain trends on the two faces of the panel provided clearer insight into the out-of-plane deformation behaviour.

**Figure 2:**
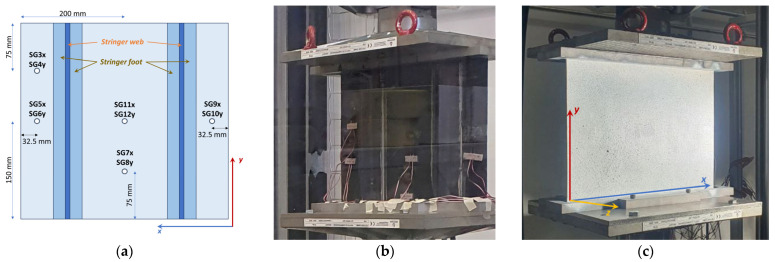
(**a**) Strain gauges locations; (**b**) panel equipped with strain gauges; and (**c**) speckle pattern.

**Figure 3:**
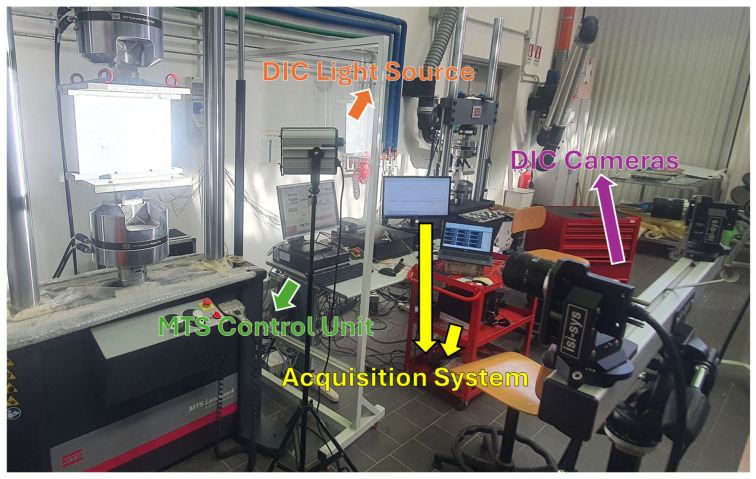
Experimental setup.

The locations of the strain gauges are illustrated in Figure 2a, and a picture of the panel’s front side, equipped with strain gauges, is shown in Figure 2b. Furthermore, the speckle pattern on the panel’s back side, used to register the information from the DIC, is shown in Figure 2c, and the experimental setup is displayed in Figure 3.

As can be observed in Figure 3, the experimental setup consists of a control unit, used to manage the MTS Landmark hydraulic testing machine; the acquisition system of the strain gauges (StrainSmart D8000, Vishay Precision Group, Chesterbrook, PA, USA), which is also connected to the MTS control unit in order to synchronize the load and displacement information taken by the testing machine with the strain gauge measurement; and the DIC equipment. In particular, the optical system used for the DIC analysis consists of two Basler ace acA2440 cameras (Basler AG, Ahrensburg, Germany) mounted on a calibrated stereo rig and controlled using VIC-3D commercial software (v9 Build 3224, Correlated Solutions Inc., Columbia, SC, USA), which is based on subset-based digital image correlation and iterative optimization procedures for displacement field evaluation. Sub-pixel accuracy is achieved through interpolation schemes, while the solution is obtained via iterative correlation algorithms (e.g., Newton–Raphson-type approaches) [37,38]. The cameras are positioned approximately 800 mm from the specimen, with a convergence angle of about 25° to ensure optimal coverage of the region of interest. The DIC images are acquired quasi-statically at selected load steps: 60 kN, 80 kN, 90 kN, 100 kN, and 115 kN. This acquisition strategy is adopted to capture the progressive strain evolution during the loading process, including the onset and development of local buckling phenomena. The DIC processing unit is not shown in this figure.

During the test, the load and strains were acquired at a frequency of 10 Hz. For safety reasons related to the structural collapse of the panel, the DIC acquisitions for loads exceeding 115 kN were halted, and only strain gauge acquisitions were carried out. The DIC processing parameters were set to a subset size of 61 and a noise level of 8, as recommended by the VIC-3D software. The analysis was carried out in 15 steps, and the calibration transformation parameters were automatically determined by the software. The study focused on the evaluation of the load–displacement response, out-of-plane displacements, strain evolution obtained from both the DIC and strain gauges, and the identification of buckling modes and damage initiation predicted by the numerical model.

## 3. Numerical Model

The numerical model was discretized in an ABAQUS environment, using continuous shell elements (SC8R). The stringers were fixed to the skin via tied constraints. To simulate the compressive load, one edge of the panel was fully constrained by fixing all translational and rotational degrees of freedom, to be consistent with the potting employed in the experimental setup. Similarly, on the opposite edge, all degrees of freedom were constrained except for the displacement in the loading direction (y-axis), where a compressive displacement was applied. The intralaminar damage mechanisms were modelled using Hashin’s failure criteria. A mesh convergence analysis was preliminarily carried out, taking into account the eigenvalue buckling load, since the present study is focused on the instability response of the panel using the predicted buckling eigenvalues and the computational time as the reference parameters. In particular, four different mesh densities were considered, including two coarser configurations and a finer one in addition to the adopted mesh. The results of the mesh convergence analysis are detailed in Table 2, where the eigenvalue and the computational time are normalized with respect to the selected mesh configuration.

**Figure 4:**
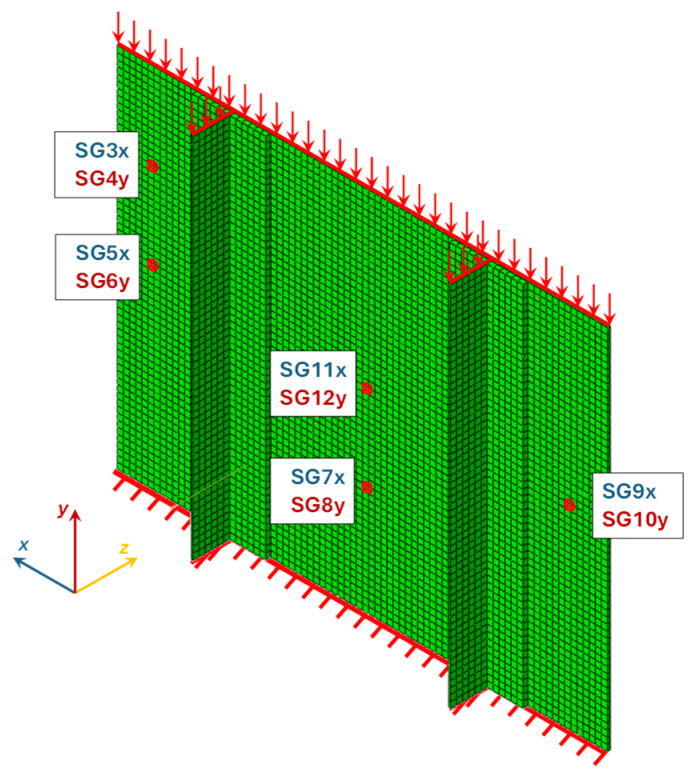
Strain gauges locations.

According to Table 2, the difference between the adopted mesh and the finer one was approximately 0.12%, while the corresponding computational time increased by about 82.6%. Therefore, the mesh consisting of 14,762 nodes and 7080 elements was adopted for all the subsequent analyses. Figure 4 shows the FEM model, including the boundary conditions and the positions of the strain gauges. The interface between the stringers and the skin was modelled using tie constraints, assuming a perfectly bonded condition. This approach does not allow for the simulation of interlaminar damage; however, it was considered appropriate for the scope of the present study, which focuses on the global buckling and post-buckling responses. Indeed, the experimental test indicated that damage initiation was primarily governed by intralaminar mechanisms, while interlaminar effects were not found to play a dominant role in the overall structural response.

## 4. Results and Discussion

**Figure 5:**
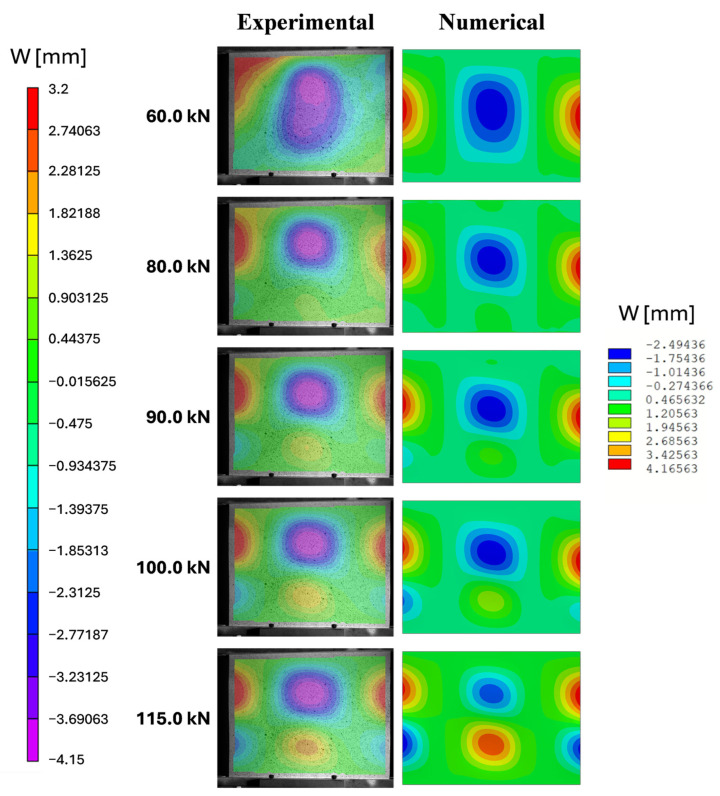
Comparison between DIC and FEM in terms of Uz contour plot.

In addition to the qualitative observations, the correlation between the experimental and numerical data was determined out using statistical indicators to provide an unbiased measure of consistency. Figure 5 compares the out-of-plane displacement field predicted by the numerical model with the corresponding displacement measured via stereo-DIC. The experimental out-of-plane displacement field exhibited a certain degree of asymmetry. This behaviour can be attributed to unavoidable experimental factors, such as a slight misalignment of the loading system and non-perfect parallelism of the loading heads, as well as geometric imperfections introduced during manufacturing and potting. These effects, although limited, can significantly influence the instability response of thin-walled composite structures, leading to deviations from the ideal symmetric behaviour predicted by numerical models.

Notably, a change in the buckling shape between 60 and 90 kN was observed. Experimentally, this buckling change occurred earlier compared to the numerical model’s prediction. This discrepancy can be attributed to imperfections within the potting, which affected all the results, particularly the strain measurements of the upper part of the panel. In the experimental test, the central wave rose more rapidly than predicted by the FEM, resulting in an earlier onset of bending effects that impacted the strains in that region.

**Figure 6:**
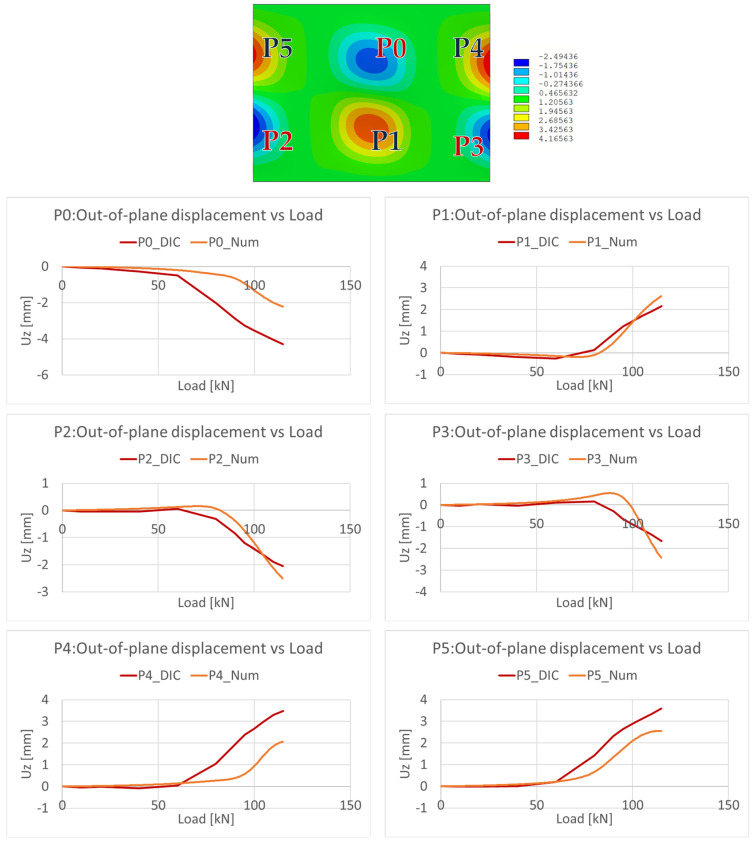
Out-of-plane displacements comparison.

In addition to the observed buckling behaviour, further insight into the experimental and numerical behaviours was gained through the analysis of the out-of-plane displacements. Specifically, the out-of-plane displacements were extracted and plotted from the DIC system at specific points, notably the centre points of the experimental buckling waves. These displacement data were then compared with the numerical outputs obtained from the corresponding locations. These comparisons are shown in Figure 6, where P#_DIC are the values extracted from the DIC system, while P#_Num are the corresponding FEM predicted values.

The results reveal a consistent trend between the out-of-plane displacements measured at the experimental and numerical points, particularly at locations P1, P2, and P3, located at the bottom of the panel. This alignment suggests that the numerical model adequately captures the observed displacements in this region, reflecting a consistent understanding of the panel’s behaviour. On the other hand, a discrepancy emerges when considering the displacement measurements at points P0, P4, and P5, located at the top of the panel. Here, the experimental test anticipates the displacements to rise more rapidly than that predicted by the numerical model. The deviation observed at points P0, P4, and P5 for load levels above 60 kN is attributed to the earlier development of the upper buckling wave in the experimental test, which led to a faster increase in the out-of-plane displacements in this region compared to the numerical prediction (Figure 5); this behaviour was mainly due to the high sensitivity of the structure to imperfections and boundary condition deviations not accounted for in the numerical model.

**Figure 7:**
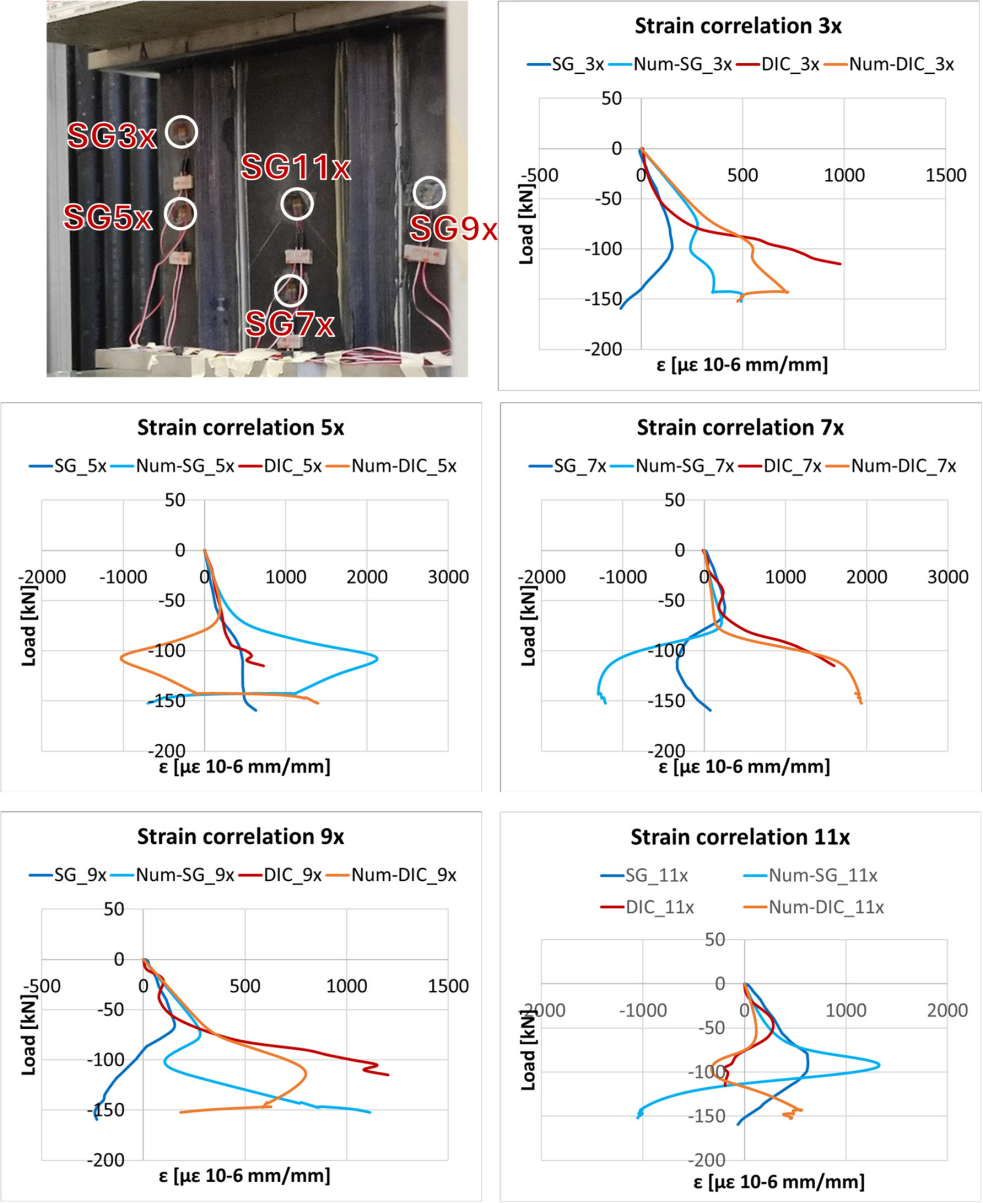
Numerical and experimental strain vs. load comparison in the x-direction (normal to the load).

**Figure 8:**
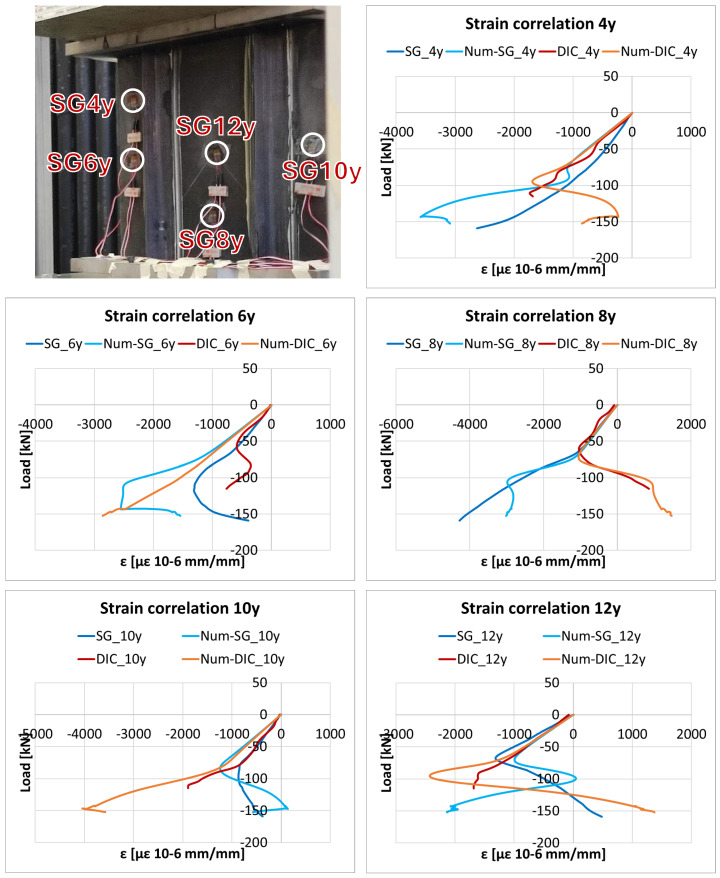
Numerical and experimental strain vs. load comparison in the y-direction (parallel to the load).

This behaviour is also reflected in the comparison of the measured and predicted strains. First, in Figure 7 and Figure 8 the experimental strains in the x- (normal to the load) and y- (parallel to the load) directions are respectively reported. In particular, the strains on the back of the panel are extracted from the strain gauge measurements (indicated as SG_#x and SG_#y), while those on the front of the panel are extracted from the DIC results (indicated as DIC_#x and DIC_#y). This dual-source comparison enables the identification of strain inversion effects associated with global buckling, and underscores the consistency and reliability of the experimental results obtained through the distinct measurement techniques. Moreover, Figure 7 and Figure 8 compare the experimental and numerical strains (indicated as Num-SG_# and Num-DIC_#), respectively, in the x- and y-directions.

According to the visual comparisons, the numerical model is able to capture the overall trend of the experimental response, particularly at points located at the bottom of the panel. In this region, the simulated displacements and strains remain consistent with the experimental data up to relatively high load levels. At the top of the panel, however, the discrepancies become more pronounced as the load increases, primarily due to imperfections and boundary effects not accounted for in the numerical model. The increasing discrepancy between the strain gauge and DIC measurements at load levels above 60 kN can be attributed to the transition to a more pronounced post-buckling regime, where the structural response becomes highly sensitive to local imperfections and boundary condition deviations. In this regime, localized measurements (strain gauges) and full-field measurements (DIC) capture different aspects of the non-uniform deformation field, leading to a progressive divergence that is not fully reproduced by the idealized numerical model.

The obtained results are consistent with a previous study on stiffened composite structures [4], which highlights the strong sensitivity of post-buckling behaviour to imperfections and boundary conditions. Both studies show the development of localized deformation patterns and a progressive divergence between the experimental observations and idealized numerical predictions at higher load levels, especially in regions influenced by stiffeners.

To provide an objective assessment of numerical–experimental agreement, a statistical analysis was carried out using standard goodness-of-fit indicators, including Pearson’s correlation coefficient r, the coefficient of determination R2, the root mean square error (RMSE), the mean absolute error (MAE), and the residual RMSE [39,40,41]. In these comparisons, two load intervals were considered: the pre-buckling and early post-buckling regimes (up to 70 kN, shown in Table 3) and the complete load history up to failure (reported in Table 4).

In the initial load range, almost all of the strain gauge (SG) measurements, based on 186 data points, exhibit a very strong correlation with the numerical predictions (r > 0.99, R2 > 0.98, RMSE < 250 µε, and residual RMSE < 30 µε). The DIC dataset, derived from only five valid points within the measurement field, is characterized by a higher scatter (0.92 ≤ r ≤ 0.99), while keeping a consistent overall trend. For the entire load history (289 points for SG, 12 for DIC), the correlation decreases and errors increase due to the discrepancy previously discussed. Indeed, the indicators reported for this range should be considered as descriptive metrics of coherence rather than a direct measurement of model accuracy, since the increasing nonlinearity of the experimental response and, especially for the DIC data, the limited number of valid measurement points intrinsically reduce the statistical correlation, even if the model is able to reproduce the overall structural behaviour.

According to Table 4, the negative correlation observed for DIC5x can be attributed to local divergence in the post-buckling regime, where the experimental and numerical trends differ due to sensitivity to imperfections and boundary conditions. A similar behaviour explains the low correlation observed for SG9x over the entire load range.

**Figure 9:**
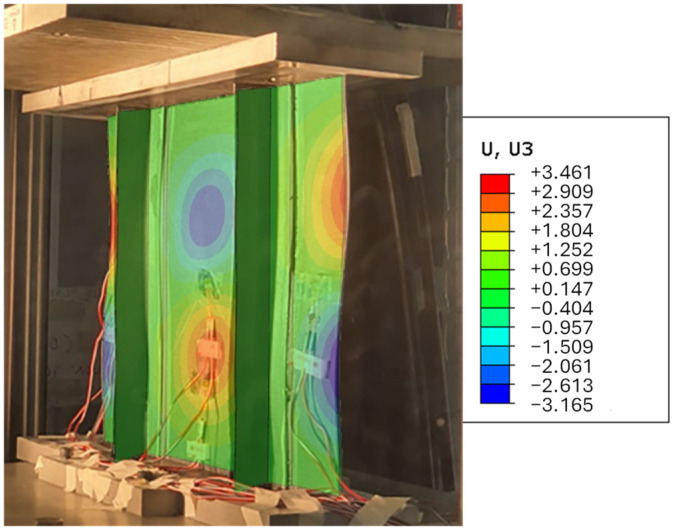
Superposition of the predicted out-of-plane displacements on the experimental deformed shape.

Despite the local discrepancies observed in the post-buckling regime, the numerical model successfully reproduces the overall post-buckling deformation shape measured experimentally, as illustrated in Figure 9. Indeed, the dominant instability mode is correctly captured, even though its onset is delayed in the numerical simulation.

**Figure 10:**
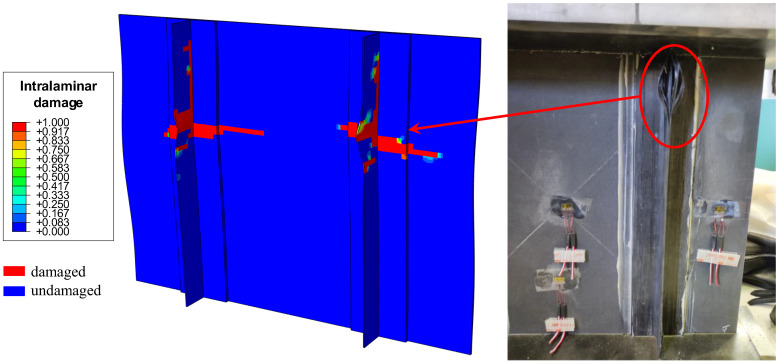
Predicted and experimental damage.

The damages were simulated by the numerical model using Hashin’s failure criteria. Notably, the damage tended to concentrate on the stringers, with occurrences of damage appearing at the stringer foot as well as on the web, as shown in Figure 10.

Figure 10 shows a contour plot of the numerical predicted failure index, describing the magnitude and distribution of damage within the panel. Here, a value of 0 is assigned to undamaged elements, while a value of one indicates complete damage of at least one of the failure mechanisms (fibre tension, fibre compression, matrix tension, and matrix compression), as per Hashin’s failure criteria. Experimentally, damage also appeared near the upper region of the panel, aligning with the earlier rise of the central wave. The observed discrepancies underscore the sensitivity of the structure to manufacturing imperfections and boundary condition inaccuracies.

Overall, the combined use of full-field displacement and strain measurements together with local strain gauge data provides a robust experimental basis for validating nonlinear finite element models of composite stiffened panels. The results demonstrate that the numerical model accurately captures the global instability behaviour and the pre-buckling response, while the local deviations in the post-buckling regime highlight the limitations of idealized assumptions when modelling real structures. Indeed, the discrepancies in some regions of the panel highlight the importance of taking real-world imperfections into account when simulating and testing composite structures.

## 5. Conclusions

An experimental–numerical investigation of the buckling and post-buckling behaviour of a reinforced composite panel subjected to compressive loading was presented. The study combined full-field displacement and strain measurements with local data from strain gauges in a back-to-back configuration, providing a comprehensive experimental characterization of both the global instability modes and local strain evolution. The experimental results were used to validate a nonlinear finite element model that included intralaminar damage based on Hashin’s failure criteria. The key response parameters analysed included the load–displacement behaviour, out-of-plane displacements, strain evolution, and damage initiation, enabling a consistent comparison between the experimental and numerical results.

The comparison between the experimental measurements and numerical predictions showed that the numerical model accurately reproduced the pre-buckling response and global deformation trends in the early post-buckling phase. In particular, a strong agreement was observed in the lower region of the panel, where both the out-of-plane displacements and deformations showed a close correspondence with the numerical results over a wide load range; the quantitative indicators confirmed this agreement as well.

A consistent difference was found in the upper region of the panel, where buckling and related bending effects occurred at lower load levels than predicted by the numerical model. This early buckling led to an early reversal of deformation and amplified local deformations on the test specimen. The analysis showed that these differences were mainly attributable to manufacturing-related geometric imperfections and uncertainties in the boundary conditions, which are difficult to characterize and were not explicitly included in the numerical model. Despite these local deviations, the numerical simulation successfully captured the dominant post-buckling deformation mode observed experimentally. These findings highlight the strong sensitivity of the instability response to even minor deviations from ideal boundary conditions, particularly in the post-buckling regime.

The damage predictions obtained using Hashin’s failure criteria indicated that damage initiation and evolution were mainly concentrated in the stringers, particularly in the area of the stringer feet and central zones. The experimental observations confirmed the occurrence of damage in similar areas, especially in the zones affected by the bending induced by the initial instability. This correspondence highlights the strong correlation between the deformation modes induced by instability and the damage mechanisms in reinforced composite panels. It is worth noting that the damage discussed in this work refers to intralaminar mechanisms, consistent with the adopted modelling approach.

Overall, the results demonstrate that the combined use of full-field measurements and local deformation data provides a robust framework for validating the use of nonlinear finite element models for instability problems. While idealized numerical models are capable of reliably capturing global instability modes and pre-buckling behaviour, the accurate prediction of local post-buckling responses requires careful consideration of imperfections and boundary effects. The results of this study highlight the importance of integrating advanced experimental measurements with numerical modelling to improve the predictive capability of simulations for real composite structures operating in post-instability regimes.

## Figures and Tables

**Table 1:** Material properties.

Property	Symbol	Value	Test
Longitudinal Young’s Modulus	E_1_	122,000 MPa	ASTM D3039 [34]
Transverse Young’s Modulus	E_2_ = E_3_	6265 MPa	ASTM D3039
Longitudinal Poisson’s Ratio	ν_12_ = ν_13_	0.3008	ASTM D3039
Transverse Poisson’s Ratio	ν_23_	0.02	ASTM D3039
Longitudinal Shear Modulus	G_12_ = G_13_ = G_23_	4649 MPa	ASTM D3518 [35]
Fibre Compressive Strength	F_1c_	697 MPa	ASTM D3410 [36]
Fibre Tensile Strength	F_1t_	1508 MPa	ASTM D3039
Matrix Compressive Strength	F_2c_	98.76 MPa	ASTM D3410
Matrix Tensile Strength	F_2t_	20.2 MPa	ASTM D3039
Shear Strength	S	96.16 MPa	ASTM D3518

**Table 2:** Results of the mesh sensitivity analysis.

Mesh Configuration	Normalized Eigenvalue	Eigenvalue Deviation (%)	Normalized Time
Coarser mesh (0.5 of the selected density)	1.0101	+1.01	0.57
Coarser mesh (0.75 of the selected density)	1.0038	+0.38	0.65
Selected mesh	1.0000	0.00	1.00
Finer mesh (1.5 of the selected density)	0.9988	−0.12	1.82

**Table 3:** Statistical indicators up to 70 kN.

Sensor ID	r	R2	RMSE	MAE	Residual RMSE
SG3x	0.99259	0.98524	81.145	65.609	11.533
SG4y	0.99815	0.9963	223.83	171.91	22.337
SG5x	0.99825	0.99651	100.05	66.614	8.4076
SG6y	0.99941	0.99882	212.44	163.42	13.358
SG7x	0.92153	0.84921	58.917	50.939	29.422
SG8y	0.9997	0.99939	47.746	36.08	9.1378
SG9x	0.99574	0.99149	57.66	41.564	8.9064
SG10y	0.99965	0.99929	191.23	143.32	9.9706
SG11x	0.98041	0.96121	72.85	65.927	30.043
SG12y	0.99919	0.99839	170.72	123.98	14.328
DIC3x	0.96722	0.93551	87.503	71.463	24.6
DIC4y	0.99398	0.988	82.763	57.614	35.823
DIC5x	0.98541	0.97104	12.165	8.9066	12.086
DIC6y	0.98509	0.9704	163.88	125.79	55.724
DIC7x	0.92666	0.8587	75.78	53.742	15.306
DIC8y	0.99268	0.98541	107.84	99.827	40.782
DIC9x	0.92199	0.85007	62.49	45.888	37.252
DIC10y	0.99358	0.9872	146.1	118.92	35.097
DIC11x	0.95416	0.91041	93.775	64.676	12.545
DIC12y	0.99907	0.99814	52.8	43.635	15.596

**Table 4:** Statistical indicators for entire load history.

Sensor ID	r	R2	RMSE	MAE	Residual RMSE
SG3x	0.85761	0.7355	109.02	88.124	56.098
SG4y	0.95608	0.91409	306.96	217.01	168.68
SG5x	0.92228	0.8506	504.37	308.93	211.91
SG6y	0.99156	0.98319	448.01	338.52	93.826
SG7x	0.85086	0.72396	209.61	117.66	163.42
SG8y	0.98352	0.96732	171.49	102.7	169.96
SG9x	0.13836	0.019144	160.97	97.468	121.23
SG10y	0.96173	0.92492	250.37	206.24	127.1
SG11x	0.87048	0.75773	338.16	208.61	248.64
SG12y	0.68937	0.47523	390.3	256.41	300.92
DIC3x	0.9336	0.87161	189.24	149.03	76.64
DIC4y	0.77496	0.60056	406.14	269.19	359.22
DIC5x	−0.9044	0.81794	932.07	657.5	195.28
DIC6y	0.86009	0.73976	784.84	644.77	320.49
DIC7x	0.97402	0.94872	167.19	126.56	146.47
DIC8y	0.98847	0.97707	199.3	170.02	101.16
DIC9x	0.98746	0.97508	232.03	180.56	46.834
DIC10y	0.9882	0.97655	382.44	298.6	137.46
DIC11x	0.8351	0.69739	111.29	90.118	89.342
DIC12y	0.87298	0.7621	470.54	350.19	416.69

## Data Availability

The original contributions presented in this study are included in the article. Further inquiries can be directed to the corresponding author.

## Abbreviations

The following abbreviations are used in this manuscript:

CFRP	Carbon Fibre-Reinforced Polymer
DIC	Digital Image Correlation
FEA	Finite Element Analysis
MAE	Mean Absolute Error
RMSE	Root Mean Square Error
SG	Strain Gauge
